# Citizen Science: The First Peninsular Malaysia Butterfly Count

**DOI:** 10.3897/BDJ.3.e7159

**Published:** 2015-12-11

**Authors:** John-James Wilson, Shi-Wei Jisming-See, Guo-Jie Brandon-Mong, Aik-Hean Lim, Voon-Ching Lim, Ping-Shin Lee, Kong-Wah Sing

**Affiliations:** ‡ Museum of Zoology, Institute of Biological Sciences, Faculty of Science, University of Malaya, Kuala Lumpur, Malaysia; § Ecology and Biodiversity Program, Institute of Biological Sciences, Faculty of Science, University of Malaya, Kuala Lumpur, Malaysia

**Keywords:** DNA barcoding, species identification, Barcode Index Numbers, butterflies, citizen science, Malaysia, invasive species

## Abstract

**Background:**

Over the past 50 years, Southeast Asia has suffered the greatest losses of biodiversity of any tropical region in the world. Malaysia is a biodiversity hotspot in the heart of Southeast Asia with roughly the same number of mammal species, three times the number of butterfly species, but only 4% of the land area of Australia. Consequently, in Malaysia, there is an urgent need for biodiversity monitoring and also public engagement with wildlife to raise awareness of biodiversity loss. Citizen science is “on the rise” globally and can make valuable contributions to long-term biodiversity monitoring, but perhaps more importantly, involving the general public in science projects can raise public awareness and promote engagement. Butterflies are often the focus of citizen science projects due to their charisma and familiarity and are particularly valuable “ambassadors” of biodiversity conservation for public outreach.

**New information:**

Here we present the data from our citizen science project, the first “Peninsular Malaysia Butterfly Count”. Participants were asked to go outdoors on June 6, 2015, and (non-lethally) sample butterfly legs for species identification through DNA barcoding. Fifty-seven citizens responded to our adverts and registered to take part in the butterfly count with many registering on behalf of groups. Collectively the participants sampled 220 butterfly legs from 26 mostly urban and suburban sampling localities. These included our university campus, a highschool, several public parks and private residences. On the basis of 192 usable DNA barcodes, 43 species were sampled by the participants. The most sampled species was *Appias olferna*, followed by *Junonia orithya* and *Zizina otis*. Twenty-two species were only sampled once, five were only sampled twice, and four were only sampled three times. Three DNA barcodes could not be assigned species names. The sampled butterflies revealed that widely distributed, cosmopolitan species, often those recently arrived to the peninsula or with documented "invasive" potential, dominated the habitat types sampled by the participants. Data from this first Butterfly Count helps establish a baseline from which we can monitor the patterns and changes in butterfly communities in Peninsular Malaysia.

## Background

### Citizen Science

Over the past 50 years, Southeast Asia has suffered the greatest losses of biodiversity of any tropical region in the world (Gibson et al. 2011). Malaysia is a biodiversity hotspot in the heart of Southeast Asia with roughly the same number of mammal species, three times the number of butterfly species, but only 4% of the land area of Australia (Table 1). Consequently, in Malaysia, there is urgent need for biodiversity monitoring and also public engagement with wildlife to raise awareness of biodiversity loss.

Citizen science is “on the rise” globally and can make valuable contributions to long-term biodiversity monitoring (Tulloch et al. 2013), although data tends to remain underutilised (Theobald et al. 2015). Perhaps even more importantly, involving the general public in science projects can raise public awareness and promote civic engagement (Loos et al. 2015). Citizen science is well established in high-income economies where projects regularly attract thousands of participants (e.g., 52,000 people took part in the UK’s Big Butterfly Count in 2015 http://www.bigbutterflycount.org; Table 1​). In transitioning economies, such as Malaysia, citizen science is less mainstream, and efforts to engage citizens face a different set of challenges. These include lack of money, time and taxonomic skills among potential participants, but also mental, cultural and socio-economic barriers (Loos et al. 2015). Participants from high-income economies often contribute their own financial resources to citizen science activities, whereas in transitioning countries, participants may require financial support to cover the cost of materials (e.g., field guides, butterfly nets) (Loos et al. 2015). More complex factors influencing participation include low levels of interpersonal trust, civic participation and social capital among the populations of transitioning economies, and the dominance of individualistic values (Loos et al. 2015). Furthermore, corruption in transitioning economies seeds mistrust of formal, and even informal, institutions (Loos et al. 2015), and feeds apathy towards all civic activities.

Against the backdrop of these challenges, here we present data and insights from our citizen science project, the first “Peninsular Malaysia Butterfly Count”.

### Butterfly Counts

Butterflies are often the focus of citizen science projects (e.g., http://scistarter.com/blog/2012/07/summer-is-busy-season-for-butterflies-and-citizen-scientists; http://www.pierisproject.org) due to their charisma and familiarity and are particularly valuable “ambassadors” of biodiversity conservation for public outreach (http://www.thestar.com.my/News/Education/2014/10/26/The-butterfly-effect/). Butterflies are thought to react rapidly to environmental changes due to their short generation time and high mobility (McIntyre 2000), and patterns of butterfly diversity are reflected in other distantly related taxonomic groups (e.g., bats; Syaripuddin et al. 2015) making them useful indicators of environmental change and degradation. Data concerning butterfly diversity is valuable in itself, as populations of butterflies are dwindling globally (New 1997) with tropical butterflies disappearing at the fastest rates (Brook et al. 2003). The butterflies of Peninsular Malaysia have been the focus of a series of comprehensive field guides written by British naturalists, beginning with Distant in 1882–1886, and followed by four editions of Corbet and Pendlebury’s classic checklist, first published in 1934 and most recently revised by Eliot in 1992 (Wilson et al. 2013). This latest edition, and accounting for some minor taxonomic changes since publication, puts the number of butterfly species recorded in Peninsular Malaysia at 1,182 (Table 1).

## Material and methods

### Preparing for Count Day

This project builds on our experience with another ongoing citizen science project, "The School Butterfly Project", which is reported elsewhere (Jisming-See et al. 2015). When we first thought about holding a butterfly count day, we searched the internet (using Google) to find out about similar projects held in other countries and discovered "Butterfly Education and Awareness Day" (also know by the acronym BEAD). BEAD is promoted by the Association for Butterflies as the first Saturday in June of each year (http://afbeducation.org/bead/). By coincidence, in Malaysia, the first Saturday of June is the Yang di-Pertuan Agong's (King's) official birthday, a public holiday. On such occasions families often like to go out for picnics in local parks, so it seemed ideal to have our first Butterfly Count on BEAD - June 6, 2015. We restricted our project to Peninsular Malaysia (West Malaysia) to avoid complications due to the shipping overseas and because the peninsula and East Malaysia (part of the island of Borneo) are governed by a different set of wildlife laws.

**Figure 1.**
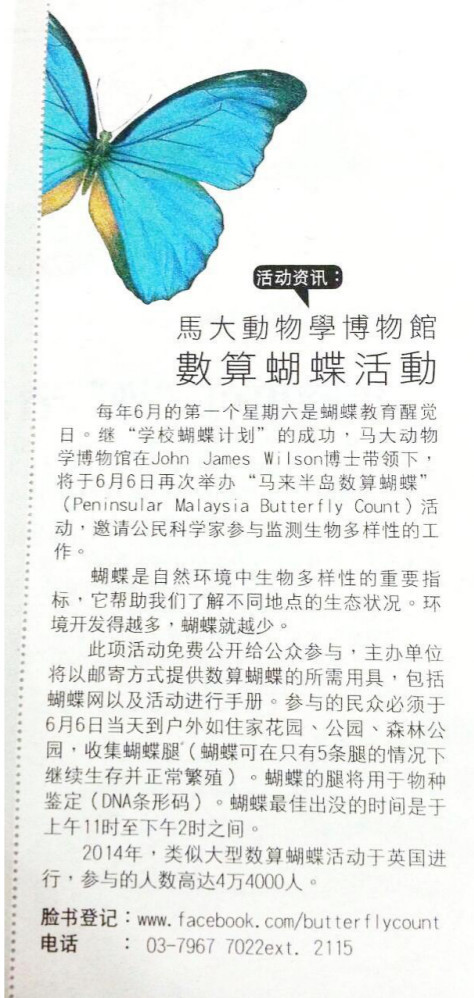
Advertisement for the first Peninsular Malaysia Butterfly Count in the Sin Chew Daily national newspaper.

As facebook is very popular in Malaysia (Table 1) the "main face" of the project was a facebook page (http://www.facebook.com/butterflycount). We created an advertisement for the project in facebook and purchased “boost post” across Malaysia to encourage maximum participation across the peninsula. According to facebook statistics the advert "reached" 27,392 people at a cost of RM73.00. We also contacted national newspapers to request coverage to encourage registration. This request was taken up by two national English-language newspapers (http://www.star2.com/living/2015/05/11/help-count- butterflies/; http://www.nst.com.my/node/83845) and one national Chinese-language newspaper (the Sin Chew Daily; Fig. 1).

Interested citizens could register online, using a Google form (in English and Malay language) linked to the facebook page, or by phone. Registration was closed on May 31, 2015.

Registered citizens were sent (via Pos Laju) a Butterfly Count Pack containing:

i) Butterfly Count Guide (Suppl. material 1), which includes details about our motivation for running the project, a brief explanation of DNA barcoding, the plan for the count day, how to make a butterfly net, how to distinguish butterfly families, how to collect butterfly legs (a video was also available on the facebook page; Fig. 2​), and a form to use on the count day.

ii) Ten 1.5ml microcentifuge tubes.

iii) Pair of tweezers.

iv) Butterfly net.

v) Prepaid addressed envelope (Pos Laju).

vi) Souvenir button badges.

Following guidelines in the Butterfly Count Guide (Suppl. material 1), citizens were asked to go outdoors on June 6, 2015, to collect butterfly legs and then mail their butterfly legs to the Museum of Zoology, University of Malaya, using the prepaid addressed envelope. Participants were also encouraged to share photographs taken on the Butterfly Count day on the Peninsular Malaysia Butterfly Count facebook page (https://www.facebook.com/butterflycount/photos_stream).

### Butterfly Identification

In September 2015, the national parks board of Singapore (NParks), also conducted an inaugural butterfly count in neighbouring Singapore (https://www.nparks.gov.sg/butterflycount). The NParks program involved a butterfly identification training workshop and assigned participants to a specified count location, requiring a significant commitment (time) and investment (travel costs) by the participants. In contrast, for the Peninsular Malaysia Butterfly Count, in order to reduce costs and encourage participation, we allowed the participants to choose their own count location, and did not provide identification training (although a simple guide to distinguish butterfly families was provided in the Butterfly Count Guide). The participants were asked to collect non-lethal tissue samples (butterfly legs) to enable accurate species identification through DNA barcoding (a DNA barcode reference library for local butterfly species has been generated previously from museum specimens; Wilson et al. 2013). Such methods have been shown to have no effect on survivorship or reproductive potential of sampled butterflies (Crawford et al. 2013, Koscinski et al. 2011, Marschalek et al. 2013) and have been used previously in Peninsular Malaysia for butterflies surveys (Syaripuddin et al. 2015, Sing et al. 2015). This method also has the advantage of providing a more personal interaction with the butterflies, matching the project objective, rather than providing dubious identifications of species "on the wing". The family-level identifications, when attempted by the participants, were compared to those obtained by DNA barcoding.

### DNA Barcoding

Genomic DNA was extracted from butterfly legs using a modified alkaline lysis method whereby legs were digested in 17.5 µl alkaline buffer for 20 minutes before adding 32.5 µl of neutralization buffer (following Ivanova et al. 2009). The DNA extracts were diluted 1/10 in ddH_2_O prior to PCR. All the DNA extracts were used for COI DNA barcoding following standard methods with the primer pair LCO1490 and HCO2198 (see Wilson 2012) or mlCOIintF and HCO2198 (Leray et al. 2013, Brandon-Mong et al. 2015). PCR amplification was performed in a 12 µl volume containing 0.125 µl of Accura Taq (Lucigen, USA), 6.25 µl of Accura 2x buffer, 1.0 µl of dNTP, 1.625 µl of ddH2O, 1.25 µl of each primer and 0.5 µl of diluted DNA. The thermocycle profile was 120 s at 94 °C followed by 40 cycles of 60 s at 94 °C, 60 s at 40 °C, 90 s at 72 °C, and a final extension step for 7 minutes at 72 °C. PCR products were visualised on a 1.5% agarose gel. PCR products were sequenced by a local company (MYTACG Bioscience, Malaysia) and the resulting chromatograms edited with CodonCode Aligner (CodonCode Corp.) and BioEdit (following Wilson 2012). The COI DNA barcodes, together with collection metadata, were submitted to the Barcode of Life Data systems (BOLD; Ratnasingham and Hebert 2007). The longer COI DNA barcodes (around 500bp or longer) were assigned to species on the basis of their BIN allocations (Ratnasingham and Hebert 2013). Shorter DNA barcodes, not allocated to BINS, were assigned species names based on >97% similarity with named DNA barcodes on BOLD.

## Results

### Participation

**Figure 3.**
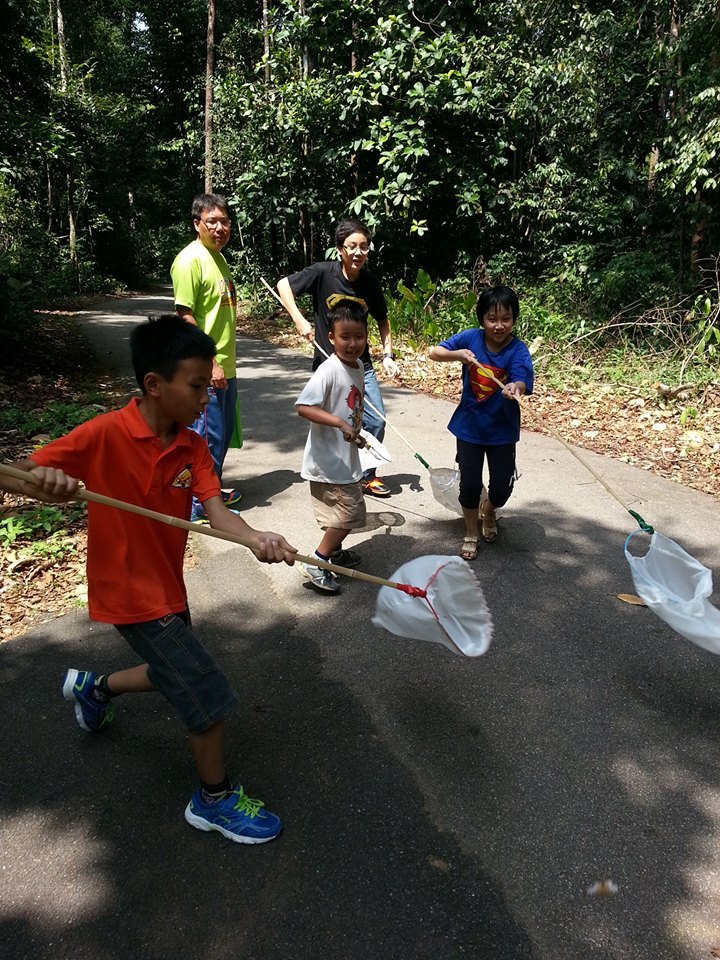
Participants on the first Peninsular Malaysia Butterfly Count day.

**Figure 4.**
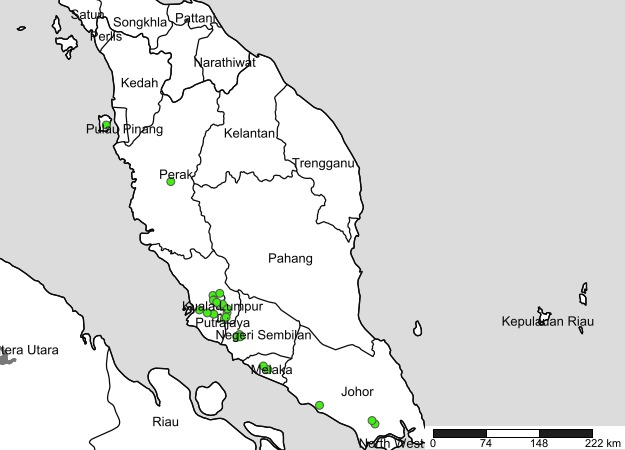
Sampling localities for the first Peninsular Malaysia Butterfly Count.

Fifty-seven citizens responded to our adverts and registered to take part in the Butterfly Count with 19 registering on behalf of groups, usually families (Fig. 3 but also one highschool. Of the 57 Butterfly Count Packs dispatched, we received 32 batches of butterfly legs (56% return rate). Some participants reported being unable to find butterflies on the count day, others told us they were afraid to collect butterfly legs and sent us photographs instead. Many required prompting via phone calls and Whatsapp messages before sending the butterfly legs to our Museum. The returned packages amounted to 220 butterfly legs from 26 mostly urban and suburban sampling localities These included our university campus, a highschool, several public parks and private residences (Fig. 4).

### DNA Barcodes

Of the 220 legs received, 192 (87%) generated 'usable' DNA barcodes of varying quality and length. The DNA barcodes and associated collection data are available on BOLD in the publicly accessible datatset - Peninsular Malaysia Butterfly Count [PMBC] (http://www.boldsystems.org/index.php/Public_SearchTerms?query=<PMBC> and dx.doi.org/10.5883/DS-PMBC).

### Butterfly Species Counted

**Figure 5.**
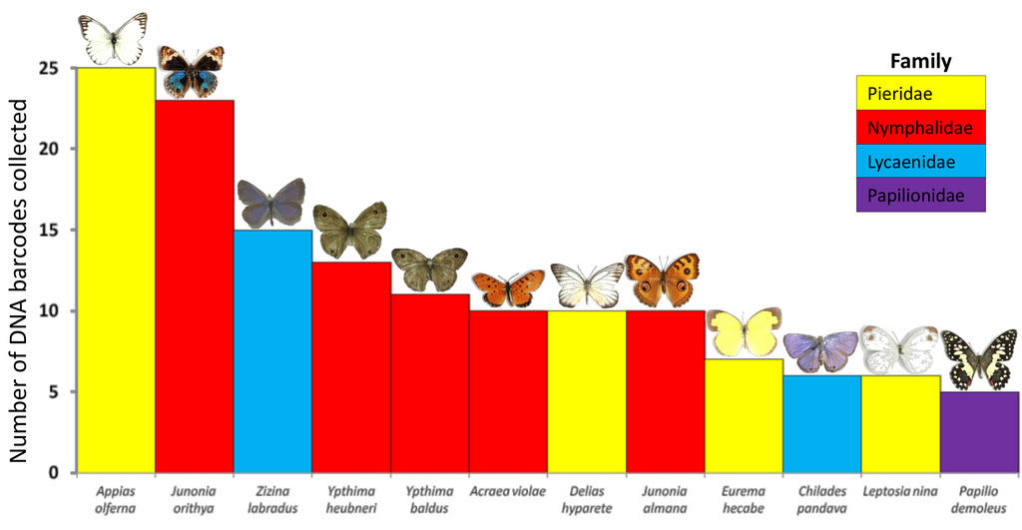
Top sampled species during the first Peninsular Malaysia Butterfly Count (Suppl. material 3).

On the basis of 192 usable DNA barcodes, 43 species were sampled by the participants. The most sampled species was *Appias olferna* (BOLD:AAZ4640), followed by *Junonia orithya* (BOLD:ABZ6191) (Fig. 5). Twenty-one species were only sampled once, four were only sampled twice, and five were only sampled three times. Three DNA barcodes could not be assigned species names. One could only be assigned to the family Hesperiidae, the two others, representing two distinct BINs (BOLD:ACW8027 and BOLD:ACX2349) were assigned to the genus *Ypthima* (Nymphalidae) using the strict tree-based criterion for DNA barcode-based higher-taxon assignment (Wilson et al. 2011).

### Family-Level Identification Success by Participants

**Figure 6.**
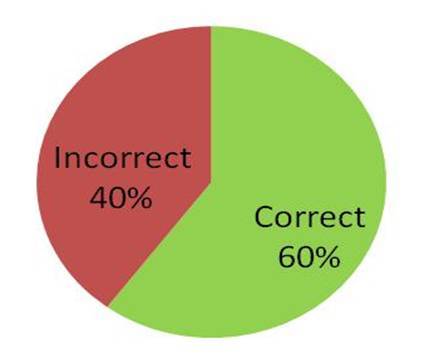
Family-level (field) identifications (108) by the first Peninsular Malaysia Butterfly Count participants compared with DNA barcode identifications (Suppl. material 3).

Of the 192 usable DNA barcodes, the participants had attempted a family-level identification for 108 (56%) of these butterflies. Based on the DNA barcode assigments, 60% of these family-level identifications were correct (Fig. 6).

### Feedback to Participants

The major findings of the Butterfly Count were collated into a newsletter (Suppl. material 2) which was posted to the Peninsular Malaysia Butterfly Count facebook page and also mailed (via Pos Malaysia) to the participants.

## Discussion

### Participation

In our initial proposal for the Peninsular Malaysia Butterfly Count, our target was to dispatch 100 Butterfly Count Packs. After registration closed we were able to send 57 packs, falling short on this target. Considering that 222 people/groups took part in the sixth annual "Malaysian Garden Bird Watch", the only comparable citizen science project in Malaysia which we are aware of (Table 1, 57 registrations in the first year of the Peninsular Malaysia Butterfly Count suggests to us a very promising start. We predicted that of the Butterfly Count Packs dispatched, roughly half would result in butterfly legs being sent to our Museum. The return rate closely matched our expectation. Several participants needed prompting before sending the collected butterfly legs, even though prepaid addressed envelopes were provided for this purpose. This was somewhat unexpected and consequently the importance of promptly returning samples will be given more prominence in any further count materials.

All of those who registered to take part in the Butterfly Count live on the west coast of Peninsular Malaysia. Furthermore, most of the participants live in the Klang Valley, the large urban agglomeration surrounding Kuala Lumpur (a similar pattern was seen with the Malaysian Garden Bird Watch; http://www.mygardenbirdwatch.com/?cur=bird/search). We need to review how to attract participation from the east coast of Peninsular Malaysia and rural areas, as it was clear we failed to reach out to those communities. Based on the registered names of participants it was also clear we were more successful in attracting participants from Malaysia's ethnic Chinese community than the other ethnic groups represented in Peninsular Malaysia. We were unable to obtain any coverage in Malay language newspapers, which may partially account for this trend, but it also likely reflects the rural-urban divide.

### DNA Barcodes and Taxonomic Identifications

Of the 220 legs received, 192 (87%) generated 'usable' DNA barcodes (i.e., with significant hits on BOLD) of varying quality and length. There are several explanations for the relatively low success rate of PCR and sequencing. As noted above, it took some butterfly legs quite a while to reach us after the count day. Depending on the storage conditions, this could have boosted DNA degradation, which is particularly a problem in hot and humid Malaysia (Wilson et al. 2013). Secondly, in order to keep the costs of the project low, and provide a sustainable model for project continuation, we used the "quick, cheap and dirty" alkaline lysis method for DNA extraction. Although, in our experience, alkaline lysis usually provides ample DNA for successful PCR, when coupled with the prolonged pre-extraction storage conditions, this could have affected the quality of the DNA extracts. Thirdly, our labwork coincided with a period of difficulty for staff at our external DNA sequencing company who are currently revising their protocols.

A comparison of the DNA barcode identifications and the family-level identifications provided by the participants revealed that the participants were able to correctly identify the family of butterfly specimens 60% of the time. The relatively low success rate suggests that our butterfly family identification guide could undergo some improvement. Preparation of an identification guide represents a trade-off between being "user-friendly" and technical, and it is important not to discourage participants from attempting identifications by providing overly-complex guidelines. The relatively low success of family identifications suggests that asking participants for species identifications would not be very useful, either as a learning experience, or for contributing data on species occurrences. This finding further validates the continued use of the non-lethal DNA barcoding model used for this project.

### The Butterflies

Although the primary purpose of the Peninsular Malaysia Butterfly Count was to promote awareness and engage the public with biodiversity, the Butterfly Count did produce some ecologically interesting findings.

The most sampled butterfly species was *Appias olferna*, commonly known as the Striped Albatross. Although most often treated as a distinct species (http://www.nic.funet.fi/pub/sci/bio/life/insecta/lepidoptera/ditrysia/papilionoidea/pieridae/pierinae/appias/index.html), *Appias olferna* is sometimes considered a subspecies of *Appias libythea* and DNA barcodes currently named as *A. libythea* and *A. olferna* share the same BIN in BOLD (BOLD:AAZ4640). *A. olferna* shows extreme sexual dimorphism with the male being predominantly white, but the female having broad black stripes on the upperside of the wings. This could potentially explain some of the family-level misidentifications by the participants, as our guide relied heavily on wing colour. According to Corbet and Pendlebury (Corbet et al. 1992), *A. olferna* was rare in Peninsular Malaysia until 60 years ago but by the early 1980s it had become one of the most common butterflies in gardens and along roadsides (Corbet et al. 1992). In Singapore, the host plant of *A. olferna* is reported to be *Cleome rutidosperma*, the Fringed Spider Flower (http://butterflycircle.blogspot.my/2010/06/life-history-of-striped-albatross.html). *C. rutidosperma* is a common weed found growing in disturbed habitats such as roadsides, gardens, and abandoned land. *C. rutidosperma* is native to Africa and is an invasive species in Peninsular Malaysia as well as other parts of Asia, Australia and the Domican Republic (http://www.cabi.org/isc/datasheet/14044#20087204071).

The second most sampled species was *Junonia orithya*, the Blue Pansy (BOLD:ABZ6191), which has a distribution covering Central Asia, India, Southeast Asia, southern China, Taiwan, Australia, and Africa (Corbet et al. 1992), and utilises a broad range of host plants. The top two most sampled species were relatively large, showy butterflies, which could be easier to spot and net by the participants. In contrast, the third most sampled species, the Common Grass Blue, *Zizina otis* (BOLD:AAB2267, also known as *Zizina labradus*; Yago et al. 2008), is a small, inconspicuous, lycaenid. *Z. otis* was the most sampled species during a survey of city parks in Kuala Lumpur (Sing et al. 2015), is one of the most abundant and widespread butterflies in Australia (New 2011), and has been considered invasive in New Zealand (Dugdale 1989). The *Ypthima* species (*Y. heubneri* BOLD:AAZ4966 and *Y. baldus* BOLD:AAN9479), at positions four and five amongst the most collected species are, together with *Eurema hecabe* (BOLD:AAA6082) at position nine, known to be paticularly common in gardens and along roadsides (Corbet et al. 1992). This is reflective of the localities where the participants conducted their sampling. The *Ypthima* group, known as the "rings", is taxonomically difficult and a review of the species in Peninsular Malaysia, including a study of the classic "ring" wing characters, is presently underway by our research group. Interestingly, two DNA barcodes collected in Penang Island (BOLD:ACW8027 and BOLD:ACX2349), fell within the *Ypthima* group of DNA barcodes on BOLD but without species-level matches. This suggests new species records for Peninsular Malaysia. At position six-equal amongst the most sampled species was *Acraea violae*, the Tawny Coster (BOLD:ABY2739, also known as *Acraea terpsicore*). The species, native to India, was first recorded in Peninsular Malaysia in 1992. The recent range expansion, reaching Australia in 2012, has been reviewed by Braby et al. 2013 who speculate on possible explanations for the expansion, including a response to climate change or habitat loss. Braby et al. 2014) also discuss the invasive potential of this species. *Chilades pandava* (BOLD:AAJ4577, also known as *Luthrodes pandava*), the tenth-equal collected species, is of concern in Taiwan and China as an "invasive" species with a history of populations "outbreaks" (Wu et al. 2010).

### Summary

1) The level of participation in the first Peninsular Malaysia Butterfly Count was encouraging, but reaching and engaging rural communities remains a challenge.

2) The non-lethal DNA barcoding approach for species identification worked effectively, however, protocols could be improved to limit the number of returned samples which could not be identified. The family-level identification guide could use some improvement but provides an important educational tool for the participants.

3) The sampled butterflies revealed that widely distributed, cosmopolitan species, often recently arrived to the peninsula or with documented "invasive" potential, dominate the habitats sampled by the participants. Data from the first Butterfly Count helps establish a baseline from which we can monitor changes in butterfly communities in Peninsular Malaysia.

## Supplementary Material

Supplementary material 1 Butterfly Count Guide Data type: Butterfly Count Guide
File: oo_48731.pdf Shi-Wei Jisming-See, Guo-Jie Brandon-Mong, Kong-Wah Sing, John-James Wilson

Supplementary material 2 Bulletin of the Museum of Zoology (3)(4) Data type: Newsletter
File: oo_60225.pdf John-James Wilson, Shi-Wei Jisming-See, Guo-Jie Brandon-Mong, Aik-Hean Lim, Voon-Ching Lim, Ping-Shin Lee, Kong-Wah Sing

Supplementary material 3 Butterfly Count Raw Data Data type: Data spreadsheet
File: oo_63256.xlsx John-James Wilson, Shi-Wei Jisming-See, Guo-Jie Brandon-Mong, Aik-Hean Lim, Voon-Ching Lim, Ping-Shin Lee, Kong-Wah Sing

## Figures and Tables

**Figure 2.** How to collect butterfly legs for DNA barcoding. The video is also available here https://youtu.be/yebuyCYRZzs.

**Table 1.** Comparison of Malaysia and Australia in terms of demography, biogeography, and public engagement with biodiversity.

Country	Malaysia	Australia
Human population	30,608,552 (http://www.statistics.gov.my/)	23,849,269 (http://www.abs.gov.au/)
Land area (km^2^)(http://www.data.un.org)	330,803	7,692,024
World bank status in 2015 (http://data.worldbank.org)	Upper-middle-income economy (Vision to be high-income economy by 2020; http://rmk11.epu.gov.my)	High-income economy
Internet users in 2014 (http://www.InternetLiveStats.com)	20,140,125	21,176,595
Facebook users in 2012 (http://www.InternetLiveStats.com)	13,589,520	11,808.360
Butterfly species	1,182 (http://malaysiabutterflies.myspecies.org)	416 (Braby 2004)
Endangered mammal species in 2015 (http://data.worldbank.org)	70	55
National nature societymembers in 2014	4,000 (Malaysian Nature Society; http://www.mns.my)	40,000 (Wilderness Society; http://www.wilderness.org.au)
Participants in national birdcount in 2014	222 (http://www.mygardenbirdwatch.com)	9,000 (http://aussiebirdcount.org.au)
