# Supplementary material for: Citizen Science: The First Peninsular Malaysia Butterfly Count
Source: Biodivers Data J. 2015 Dec 11;(3):e7159. doi: 10.3897/BDJ.3.e7159 (PMC4700385; doi:10.3897/BDJ.3.e7159)
Supplement: Supplementary material 1 — Butterfly Count Guide [file biodiversity_data_journal-3-e7159-s001.pdf]

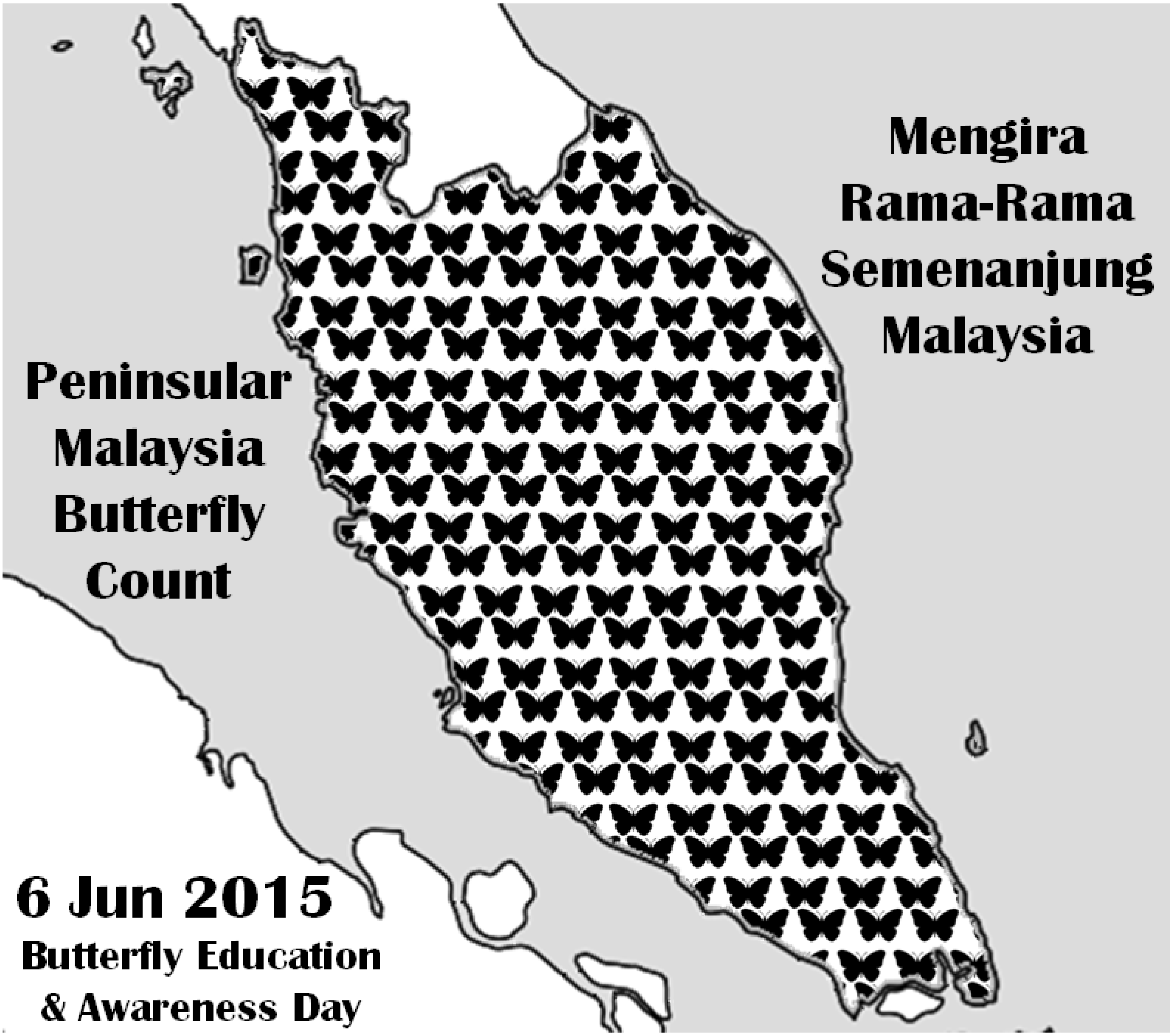

BUTTERFLY COUNT GUIDE

PANDUAN MENGIRA RAMA-RAMA

Museum of Zoology
University of Malaya

UNIVERSITI MALAYA
KUALA LUMPUR

UM Cares

Untuk pengetahuan anda, secara sainstifiknya **kupu-kupu = butterflies**; **rama-rama = moths**.

Walau bagaimanapun, masyarakat umum telah biasa menggunakan rama-rama bagi merujuk “butterflies” seperti Taman Rama-Rama yang membawa makna “Butterflies Park”. Oleh demikian, kami akan menggunakan rama-rama merujuk “butterflies” bagi mengelakkan keliruan kalangan umum.

Prepared by

Jisming See Shi Wei
Brandon Mong Guo Jie
Sing Kong Wah
John James Wilson

CONTACT INFORMATION

MAKLUMAT PERHUBUNGAN

Online:
facebook.com/butterflycount

Email: butterflycount@gmail.com

Phone: 03-7967-7022 ext. 2115

BUTTERFLY COUNT GUIDE

1. Learn about the BUTTERFLY COUNT and DNA BARCODES [See pages 8-9]

2. Plan for the BUTTERFLY COUNT DAY [See pages 10-11]

3. Make your BUTTERFLY NET [See page 12]

4. Learn how to tell which FAMILY a BUTTERFLY belongs to [See page 14]

5. Learn how to collect BUTTERFLY LEGS [See pages 16-17]

6. COUNT BUTTERFLIES

Go out on **6 June** and collect butterflies legs [Use the COUNTING FORM on page 21]

BUTTERFLY COUNT GUIDE

7. Mail your BUTTERFLY LEGS to Museum of Zoology, University of Malaya using the prepaid envelope

8. Wait for the BUTTERFLY COUNT results!

We will post the findings of the Butterfly Count on the facebook page and share with the national media. You can also call or email us to find out the results.

[See CONTACT INFORMATION page 3]

THANK YOU!

PANDUAN KIRAAN RAMA-RAMA

1. Belajar tentang KIRAAN RAMA-RAMA dan DNA BARCODES [Lihat muka surat 8-9]

2. Merancang HARI KIRAAN RAMA-RAMA [Lihat muka surat 10-11]

3. Membuat JARING RAMA-RAMA anda [Lihat Muka surat 13]

4. Belajar dan mengetahui FAMILI RAMA-RAMA [Muka surat 15]

5. Belajar bagaimana untuk mengumpul KAKI RAMA-RAMA [Lihat muka surat 18-19]

6. MENGIRA RAMA-RAMA

Keluar pada 6 **Jun** dan mengumpul kaki rama-rama [Gunakan BORANG KIRAAN pada muka surat 22]

PANDUAN KIRAAN RAMA-RAMA

7. Mengirim KAKI-KAKI RAMA-RAMA anda ke Muzium Zoologi , Universiti Malaya dengan menggunakan sampul surat prabayar

8. Tunggu keputusan KIRAAN RAMA-RAMA!

Kami akan memaparkan keputusan Kiraan Rama-rama melalui laman facebook dan media tempatan. Anda juga boleh membuat panggilan atau email kami untuk mengetahui keputusan.[Lihat MAKLUMAT PERHUBUNGAN muka surat 3]

TERIMA KASIH!

Over the past 50 years, Southeast Asia has suffered the greatest losses of biodiversity of any tropical region in the world and there is urgent need for **biodiversity monitoring**. A small group of species is used to estimate “total” biodiversity as counting all the species present in one area is impossible. Butterflies can be found all over Peninsular Malaysia and studies date back to 1882. By going out into nature and interacting with butterflies we hope that you will become engaged with the biodiversity that is currently on your doorstep but might not be there in the near future, unless collective action is taken.

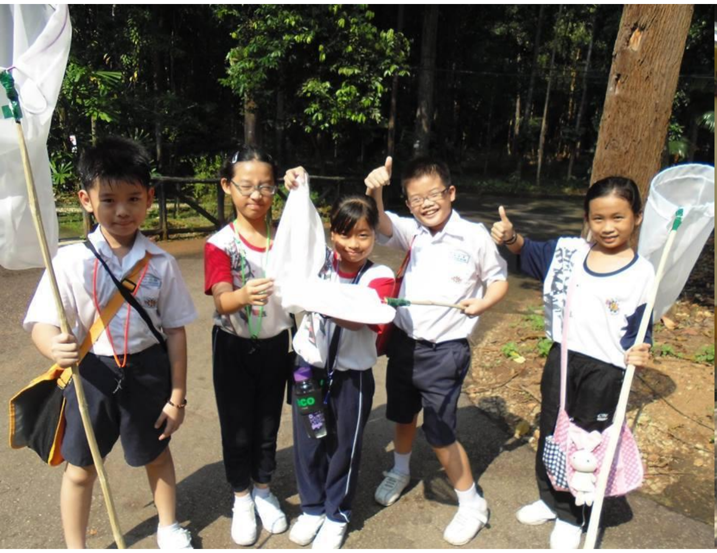

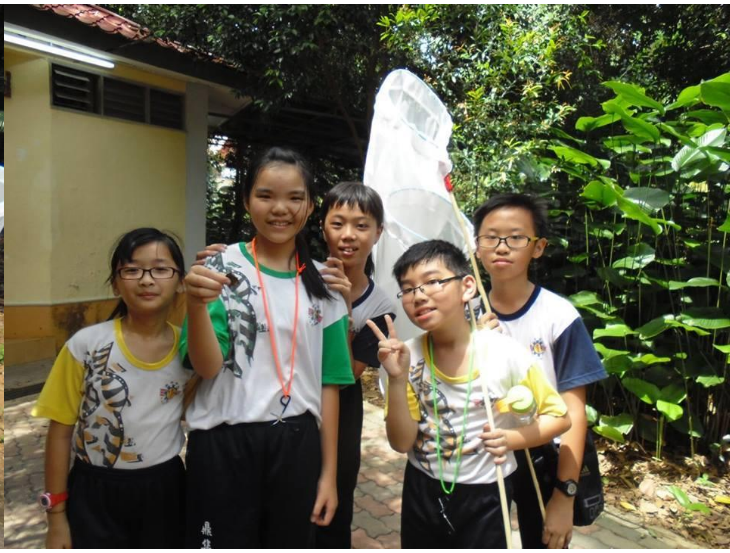

Sejak 50 tahun yang lalu , Asia Tenggara telah mengalami kehilangan kepelbagaian biologi yang terbanyak berbanding dengan rantau-rantau tropika lain di dunia. Oleh itu, pemantauan kepelbagaian biologi amat diperlukan. Sekumpulan kecil spesies digunakan untuk menganggarkan "jumlah" kepelbagaian biologi kerana mengira semua spesies yang wujud di sesuatu kawasan adalah mustahil. Rama-rama boleh didapati di seluruh Semenanjung Malaysia dan kajian rama-rama bermula sejak1882. Dengan mendekati alam semula jadi dan berinteraksi dengan rama-rama, kami berharap bahawa anda dapat melibatkan diri dengan kepelbagaian biologi yang kini di depan pintu anda tetapi yang mungkin akan hilang pada masa depan, melainkan tindakan pengumpulan sample diambil.

DNA BARCODES

Species can sometimes be hard to identify. Even butterflies. Even for experts.

By reading a small segment of an organism's DNA you have a unique identifier for the species, just like the barcodes on retail products in supermarkets.

By matching an unknown DNA barcode against a library of DNA barcodes you can identify species.

Kadang-kala, species adalah sukar untuk dikenal pasti. Mahupun rama-rama. Mahupun pakar-pakar.

Dengan membaca satu segmen kecil DNA, anda mempunyai pengenal pasti yang unik untuk sesuatu spesies, seperti kod bar pada produk runcit di pasar raya.

Dengan memadankan kod bar DNA yang tidak diketahui dengan perpustakaan kod bar DNA, anda dapat mengenal pasti spesies tersebut.

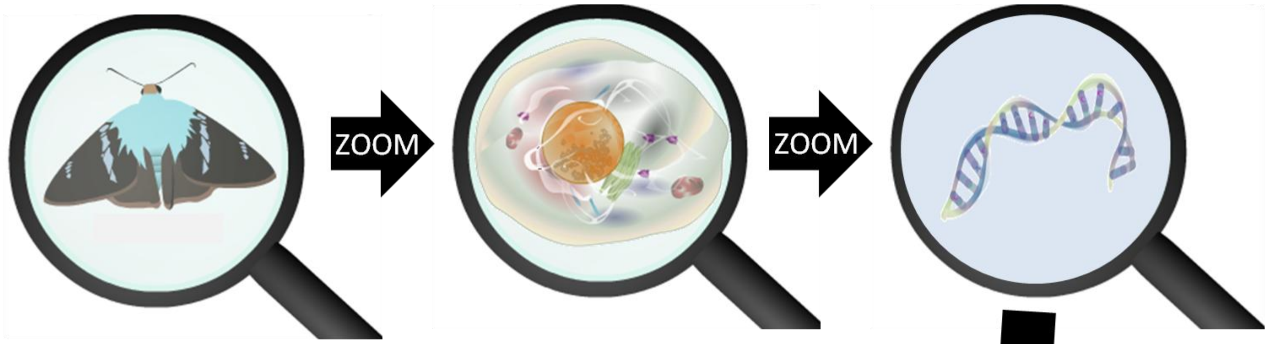

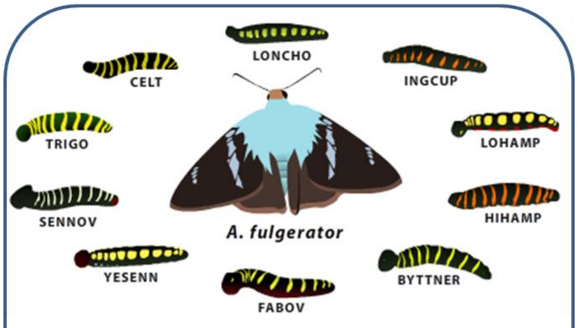

The DNA barcodes can identify the 10 different species of butterflies which look very similar as adults

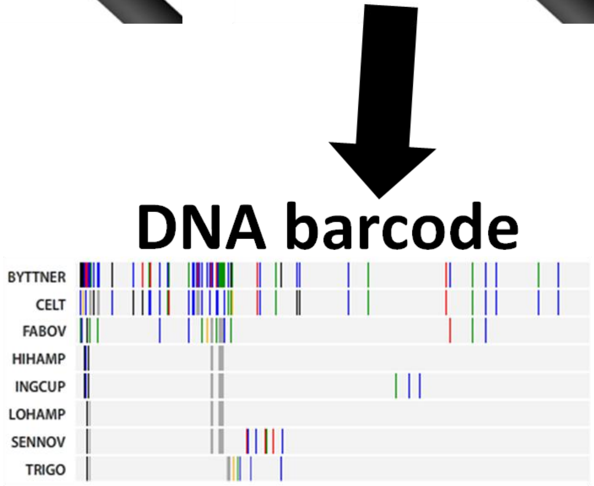

A segment of DNA unique to a species

Learn more about DNA barcodes here:
https://www.youtube.com/watch?v=ZImiXgU6bCk

PLAN FOR COUNT DAY

If you plan to count butterflies at someone else's garden, make sure you get their permission first!

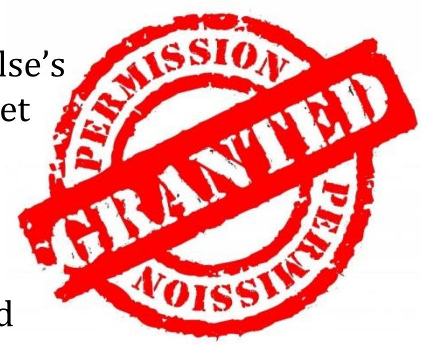

Jika anda merancang untuk mengira rama-rama di taman orang lain, pastikan anda mendapat kebenaran dahulu!

If you plan to count butterflies at a public garden, check if you need permission first - ask the park staff.

Jika anda merancang untuk mengira rama-rama di taman awam, kenal pasti sama ada kebenaran diperlukan - tanya kakitangan taman.

Some butterflies are protected species in peninsular Malaysia and should not be handled.

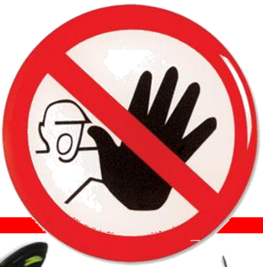

Beberapa species rama-rama adalah spesies yang dilindungi di Semenanjung Malaysia dan adalah dilarang tangkap.

Trogonoptera spp.
Troides spp.
Papilio agestor
Papilio mahadeva
Papilio palinurus
Charaxes solon
Charaxes distanti
Charaxes durnfordi
Charaxes harmodius
Charaxes borneensis

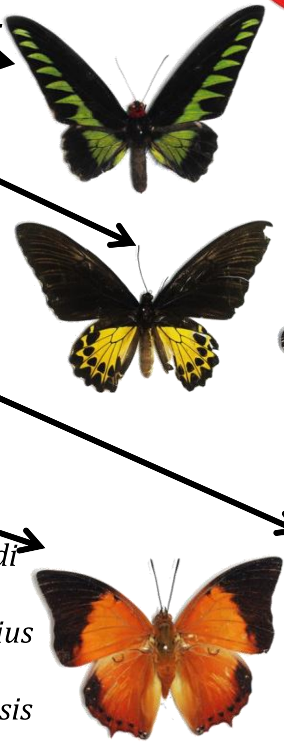

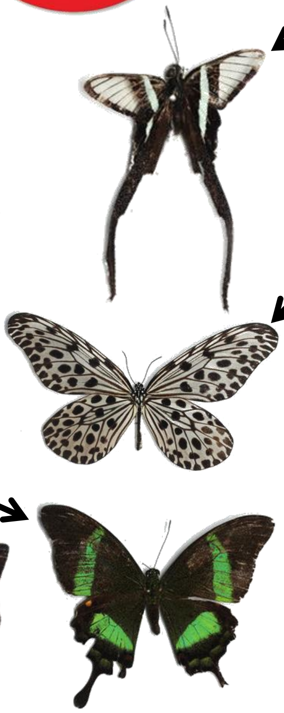

Lamproptera curius
Lamproptera meges
Danaus affinis
Idea lynceus
Idea leuconoe
Polyura schreiber
Polyura eudamippus
Zeuxidia aurelius
Enispe intermedia
Prothoe franck

But if you do see any of these species take a photo and let us know!

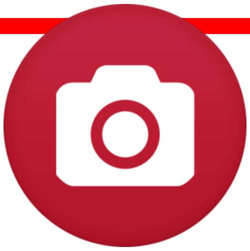

Tetapi jika anda terlihat spesies-species tersebut, sila ambil gambar mereka dan beritahu kami!

RANCANG UNTUK HARI KIRAAN

The best time to see butterflies is between 11am and 2pm

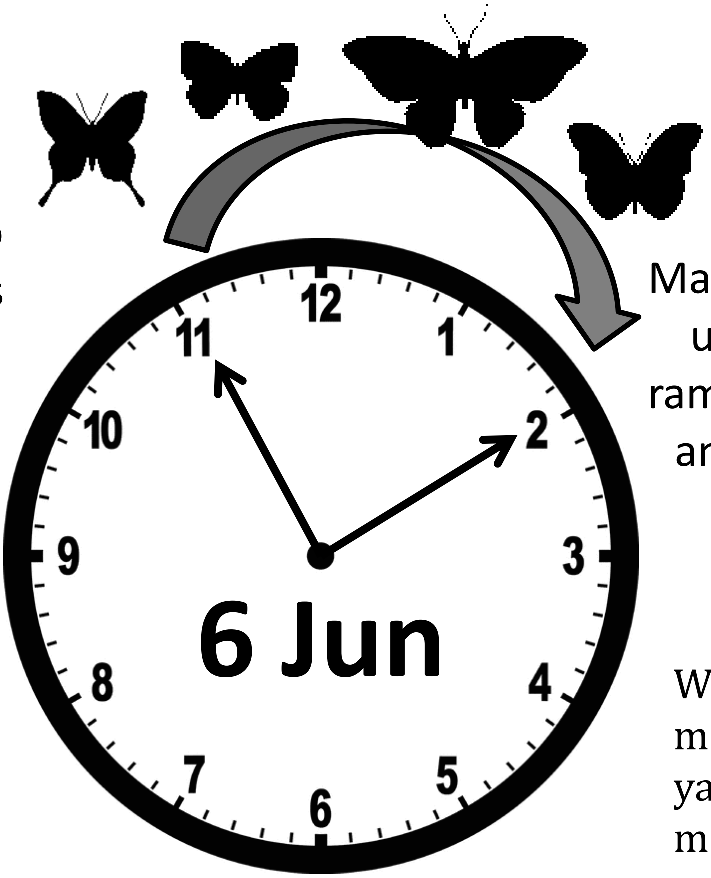

Masa yang terbaik untuk melihat rama-rama adalah antara 11am ke 2pm.

This is the hottest part of the day when the sun is at its brightest. Take precautions such as applying sunscreen, wearing a hat, drinking lots of water and using mosquito repellant.

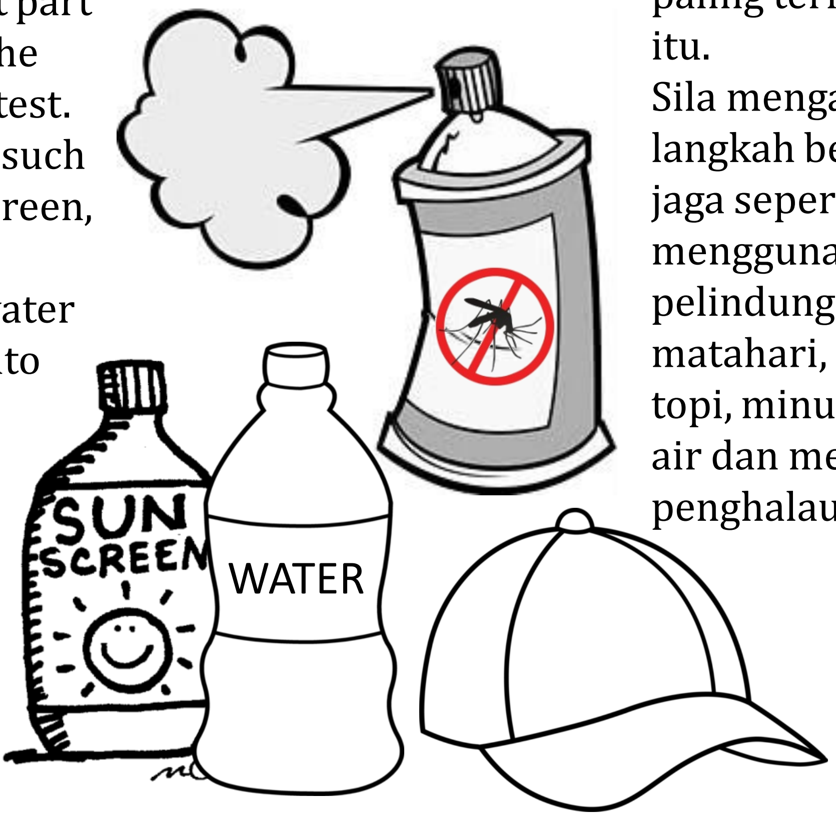

Waktu tersebut merupakan masa yang terpanas dan matahari adalah paling terit ketika itu.
Sila mengambil langkah berjaga-jaga seperti mengguna kream pelindung cahaya matahari, memakai topi, minum banyak air dan mengguna penghalau nyamuk.

MAKE YOUR BUTTERFLY NET

You will need:
-A wire clothes hanger.
-A long stick (1m long or longer. Bamboo pole is very suitable).
-A roll of heavy duty electrical tape ("duct" tape).
-The butterfly net provided in your count pack.
-Help from an adult. The ends of the wire clothes hanger can be sharp. **Remember safety first!**

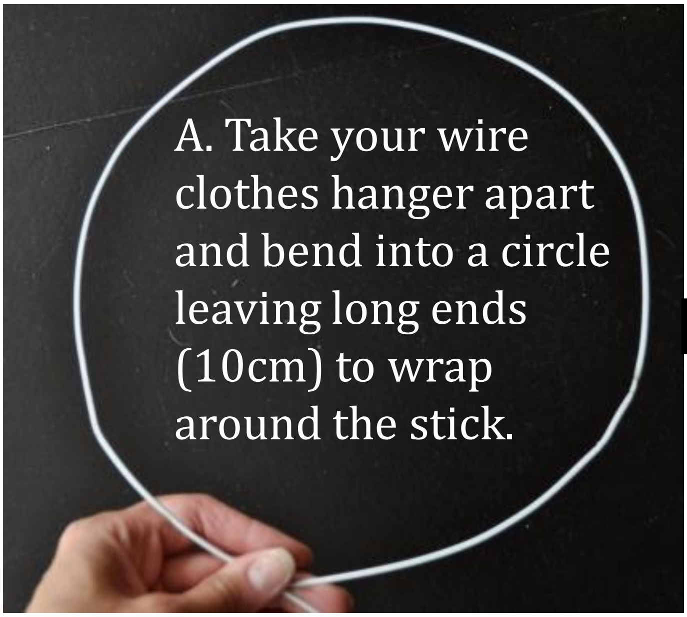

A. Take your wire clothes hanger apart and bend into a circle leaving long ends (10cm) to wrap around the stick.

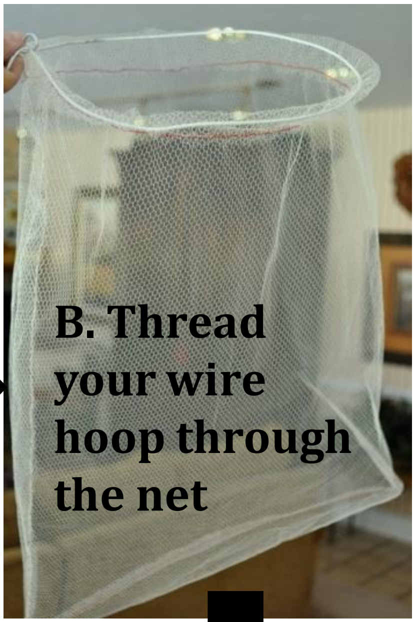

B. Thread your wire hoop through the net

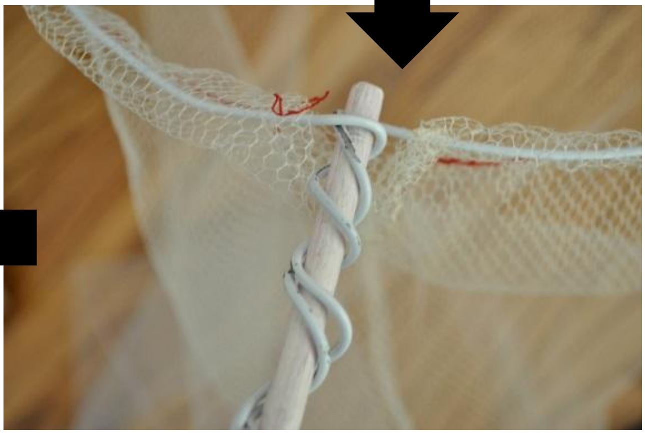

C. With your net on the hoop, wrap and twist the ends of the hanger around one end of the stick.

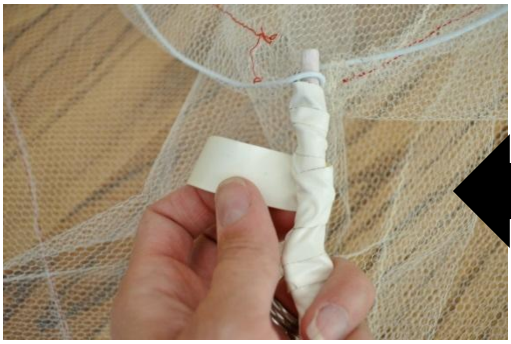

D. Cover the wire with lots of electrical tape to make it sturdy

MEMBUAT JARING RAMA-RAMA

Anda perlu:
-satu penyidai berdawai
-satu kayu panjang (1m panjang atau lebih. Batang buluh adalah sangat sesuai)
-Segulung TAPE
-Kain jaring rama-rama akan dibekalkan di dalam kiriman bungusan.
-Minta bantuan orang dewasa, hujung pengidai mungkin tajam.
Ingat, keselamatan diutamakan!

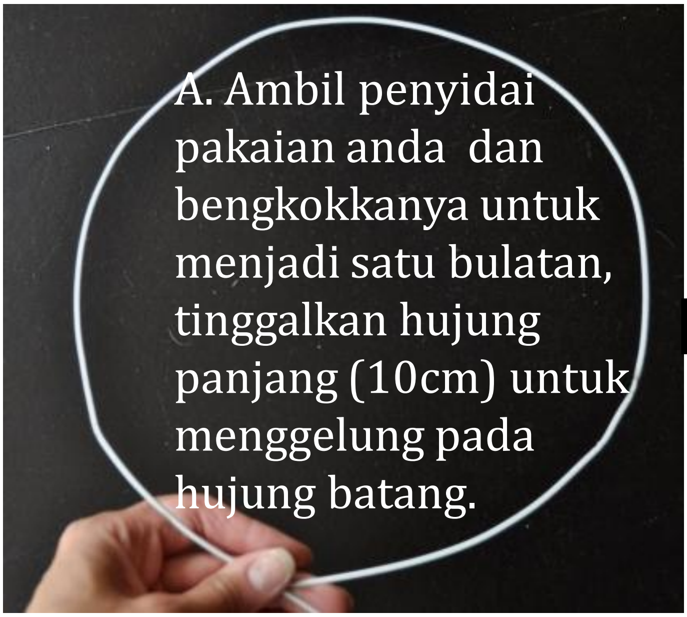

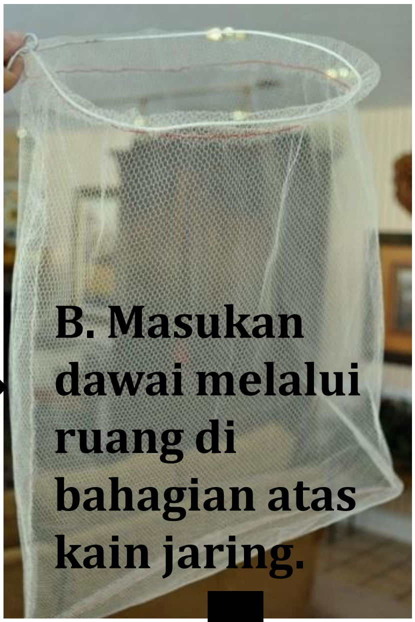

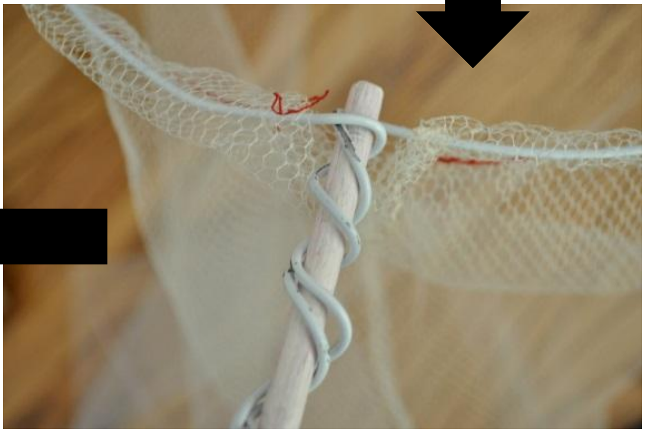

C. Selepas dawai dimasukan ke dalam kain, gelung dan pusingkan hujung dawai pada hujung batang.

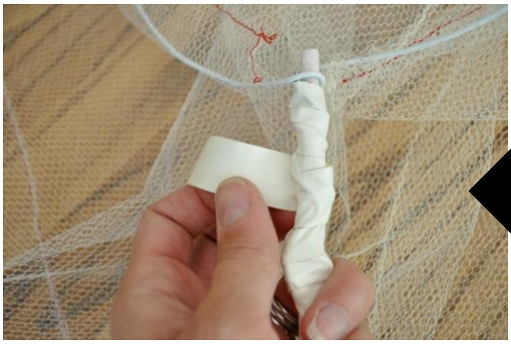

D. Balutkan dawai dengan tape untuk mengukuhkanya pada batang

BUTTERFLY FAMILIES

Butterfly or moth?

- In general, butterflies are active **at day**, moths are active **at night**.
- Moths tend to be **heavy-bodied**, butterflies are **slender**.
- The best way is to look at the **antenna**. Moths have a variety of antenna types, often with hairs.
- Butterfly antenna have no hairs but a **pronounced club** at the end so that they resemble a matchstick.

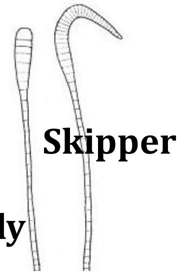

Skipper

"True" butterfly

Skipper or "true" butterfly?

The **Hesperiidae (Skippers)** are easy to distinguish from the other butterfly families, known as the "true" butterflies.

- Medium/small size
- Antenna club tapers to a point

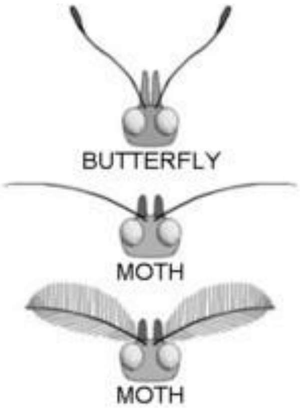

Which "true" butterfly family?

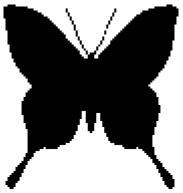

Papilionidae Swallowtails

- Large size
- Wings usually black, with red, white, yellow, blue or green markings
- 46 species in Peninsular Malaysia

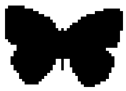

Pieridae Yellows & Whites

- Medium size
- Wings white or yellow with black markings, legs are well-developed
- 47 species in peninsular Malaysia

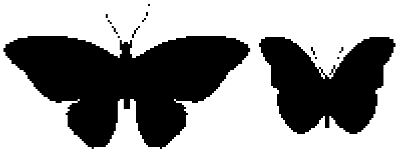

Nymphalidae Four foots

- Large/medium size
- Wings diverse in shape and colour, known as the four foots because the front pair of legs is reduced
- 282 species in peninsular Malaysia

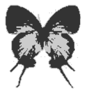

Lycaenidae Blues, Coppers & Hairstreaks

- Small size
- Wings have a powdery appearance sometimes with metallic gloss and antenna like tails
- 402 species in peninsular Malaysia

FAMILI RAMA-RAMA

Rama-rama atau “moth”?

- Secara umumnya , rama-rama aktif pada siang hari , “moth” aktif pada waktu malam.
- “moth” bertubuh berat, manakala rama-rama adalah langsing.
- Cara yang terbaik adalah melihat antena. “moth” mempunyai pelbagai jenis antena , biasanya bercabang .
- Antena rama-rama tidak mempunyai cabangan tetapi berbentuk tombol pada hujung seperti batang mancis.

Skipper atau rama-rama “benar”?

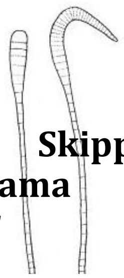

Hesperiidae (Skippers) mudah dibezakan daripada famili rama-rama yang lain, yang dikenali sebagai rama-rama "benar”.

- Sederhana / saiz kecil
- Antena bertombol dan berbentuk cangkuk memancing pada hujungnya.

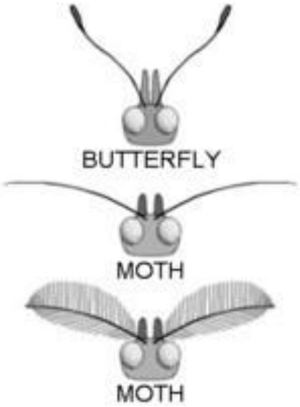

Yang manakah famili rama-rama “benar”?

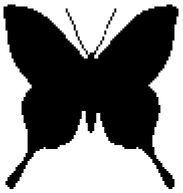

Papilionidae Swallowtails

- Saiz besar
- Kepak biasanya hitam, dengan merah, putih, kuning, biru atau hijau
- 46 spesies di Semenanjung Malaysia

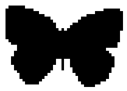

Pieridae Kuning & Putih

- Saiz sederhana
- Kepak berwarna putih atau kuning dengan corak hitam, kaki yang bertumbuh sempurna
- 47 spesies di Semenanjung Malaysia

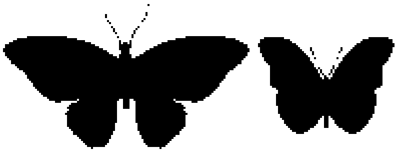

Nymphalidae Empat kaki

- Saiz besar/ sederhana
- Kepak yang mempunyai pelbagai bentuk dan warna, dikenali sebagai empat kaki kerana pasangan depan kaki terosot
- 282 spesies di Semenanjung Malaysia

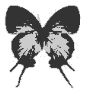

Lycaenidae Biru, Tembaga & Berjalur rambut

- Saiz kecil
- Kepak mempunyai penampilan serbuk dan kadang-kala kilauan logam dan hujung kepak berbentuk antena
- 402 spesies di Semenanjung Malaysia

HOW TO COLLECT BUTTERFLY LEGS

1. Find butterflies

Butterflies usually can be found near large patches of flowers, weeds, and grassy places.

2. Wait for the butterfly to land

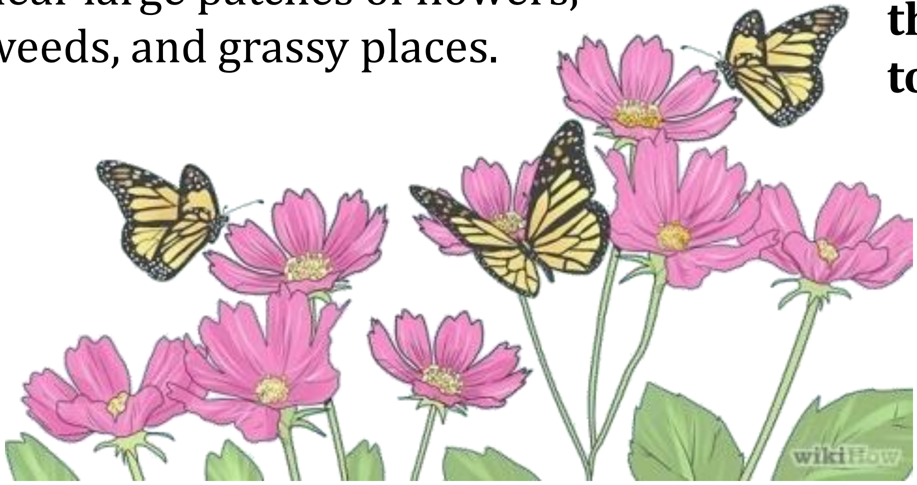

3. Catch the butterfly

Use a horizontal side-swipe with the net. Do not swipe your net at the butterflies in flight too often this will frighten butterflies away

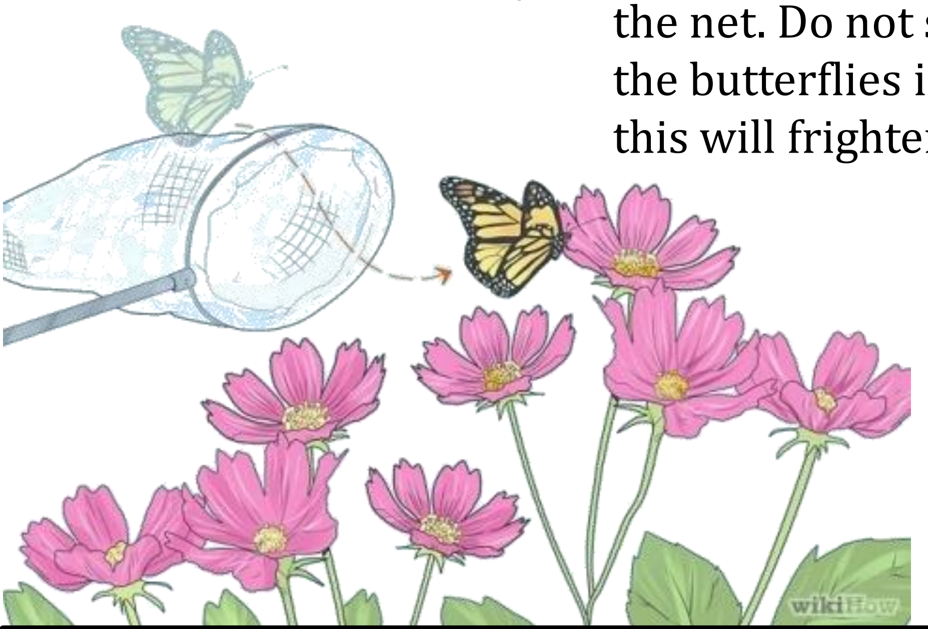

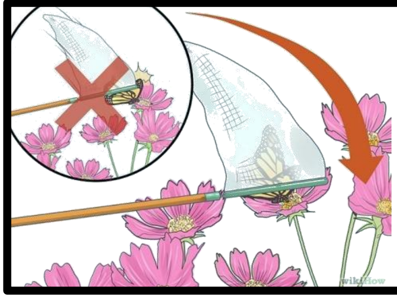

WARNING: Be careful not to hit the butterfly with the edge of your net. Try to always swing the net horizontally to the ground.

4. Gently take hold of the butterfly

Coax the butterfly into the top part of the net then reach in and gently take hold of the butterfly by holding its wings together.

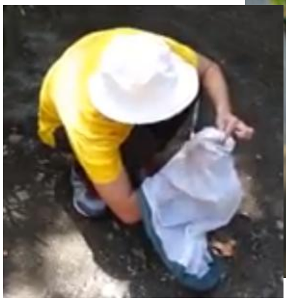

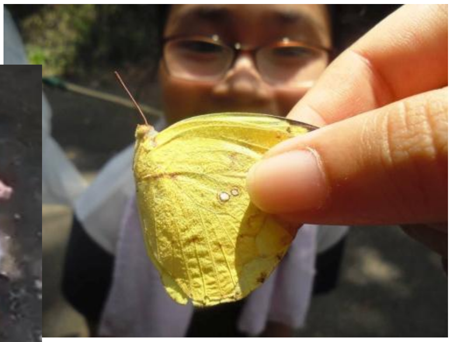

5. Take the butterfly's Left Hind Leg (LHL)

Use tweezers provided in the sampling kit, gently pull off the left hind leg. Put the leg into one of the tubes, and firmly close the cap.

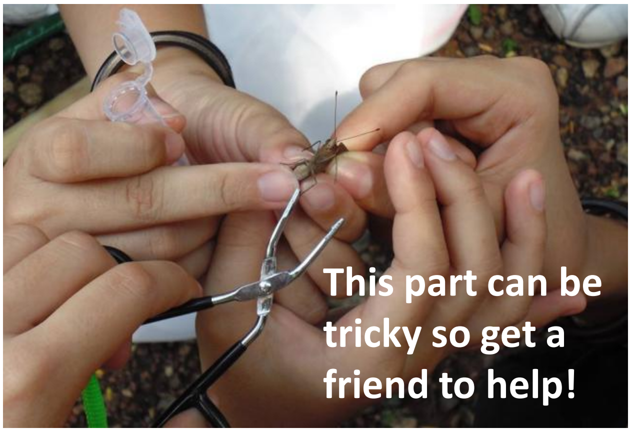

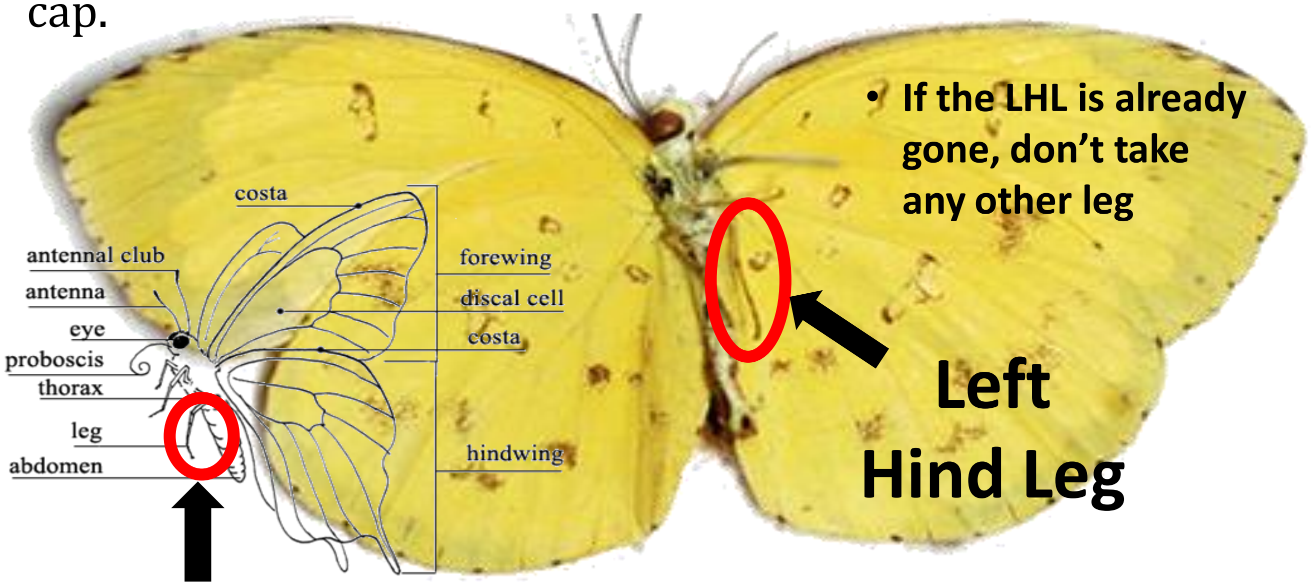

6. Release the butterfly and watch it fly away

If possible take a photo the butterfly and identify its family

WATCH the video on the facebook page for more information.
Don't hurt the butterfly, treat it with respect!

CARA UNTUK MENGUMPUL KAKI RAMA-RAMA

1. Mencari rama-rama

Rama-rama biasanya boleh dijumpai dekat bunga, rumpai, dan tempat-tempat berumput .

2. Tunggu rama-rama untuk berdarat

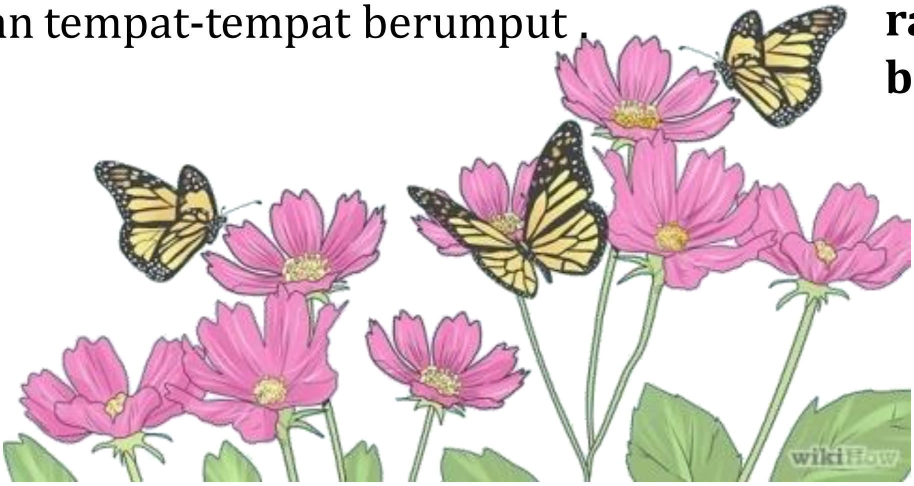

3. Tangkap rama-rama

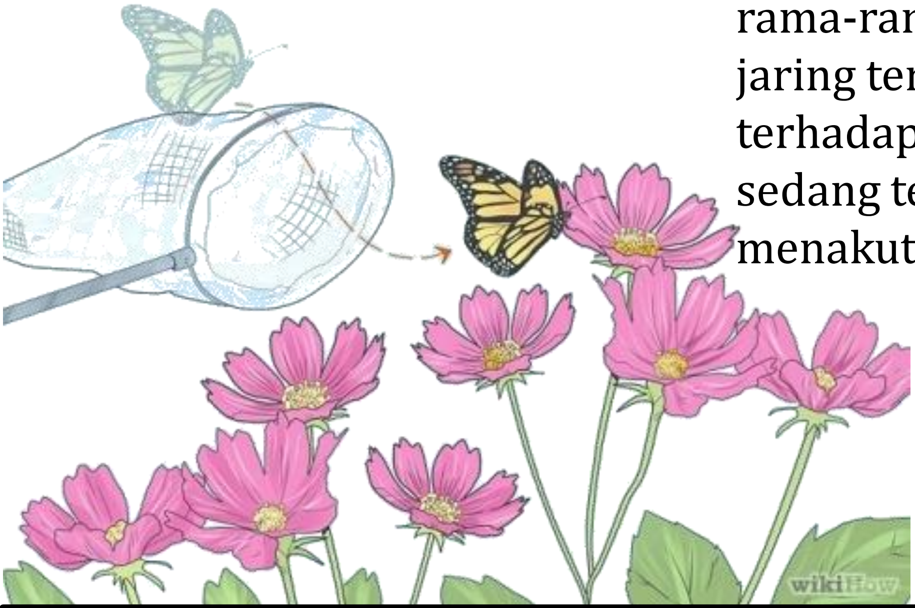

Gerakkan jaring secara mendatar semasa menangkap rama-rama. Jangan gerakkan jaring terlalu banyak kali terhadap rama-rama yang sedang terbang kerana ini akan menakutkan mereka.

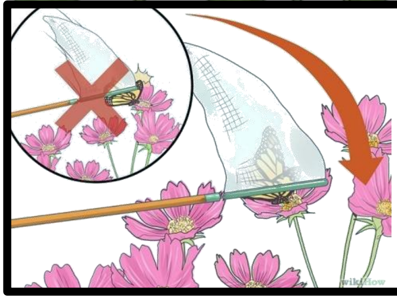

AMARAN: Berhati-hati dan jangan memukul rama-rama dengan tepi jaring anda . Cuba selalu gerakkan jaring ke tepi secara mendatar.

4. Menangani rama-rama secara lembut

Memujuk rama-rama ke bahagian atas jaring dan kepit kepak rama-rama secara lembut dengan jari anda selepas menghulur tangan anda ke dalam jaring.

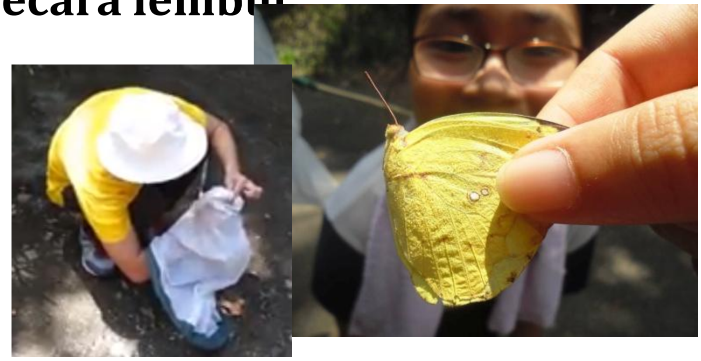

5. Ambil kaki kiri akhir (KKA)

Tarik kaki kiri akhir rama-rama dengan penyepit yang diberi dalam bungusan. Masukkan kaki tersebut ke dalam tiub dan tutup dengan ketat.

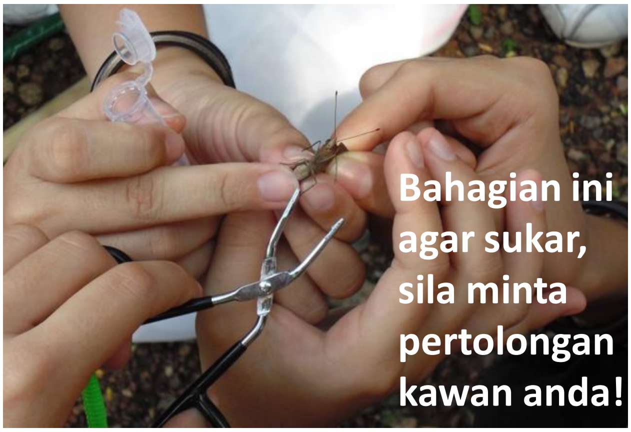

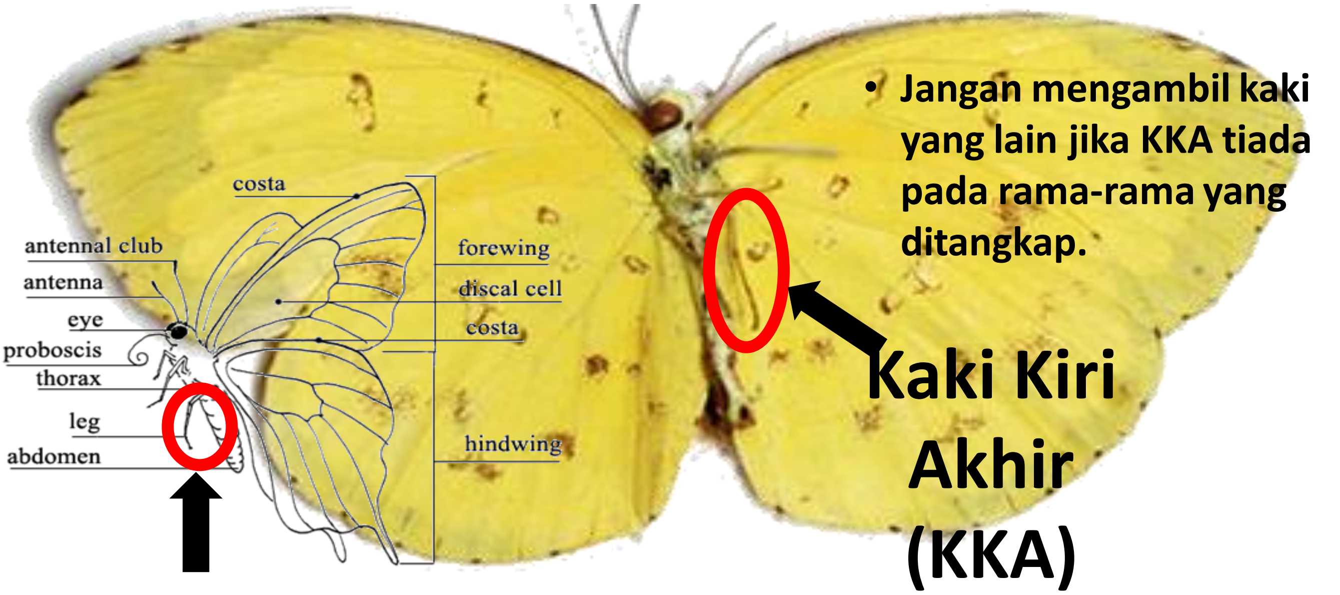

6. Lepaskan rama-rama dan lihatnya berterbang

Jika boleh, tangkap gambar rama-rama dan cuba mengecam famili-nya

Untuk maklumat lanjut, sila TONTON video di laman facebook.
Jangan mencederakan rama-rama, layan ia dengan hormat!

COUNTING FORM/BORANG PENGIRAAN

NAME/NAMA: ___________________________________

WHERE DID YOU COUNT BUTTERFLIES?/DI MANAKAH ANDA MENGIRA RAMA-RAMA?

____

**TUBE NUMBER/ NOMBOR TIUB**	**BUTTERFLY FAMILY/ FAMILI RAMA-RAMA**
1	
2	
3	
4	
5	
6	
7	
8	
9	
10	

PUT THIS COUNTING FORM IN THE PREPAID ENVELOPE WITH YOUR BUTTERFLY LEGS/MASUKKAN BORANG PENGIRAAN INI KE DALAM PRABAYAR SAMPUL SURAT DENGAN KAKI-KAKI RAMA-RAMA YANG TELAH DIKUMPUL
