# Supplementary material for: Citizen Science: The First Peninsular Malaysia Butterfly Count
Source: Biodivers Data J. 2015 Dec 11;(3):e7159. doi: 10.3897/BDJ.3.e7159 (PMC4700385; doi:10.3897/BDJ.3.e7159)
Supplement: Supplementary material 2 — Bulletin of the Museum of Zoology (3)(4) [file biodiversity_data_journal-3-e7159-s002.pdf]

# BULLETIN OF THE MUSEUM OF ZOOLOGY

## SPECIAL ISSUE: FIRST PENINSULAR MALAYSIA BUTTERFLY COUNT

The first Peninsular Malaysia Butterfly Count took place on **6 June 2015**. In this special issue we share the findings from the count.

## WHO TOOK PART?

Butterfly legs were collected at 26 locations including UM campus, a highschool, several public parks and private residences.

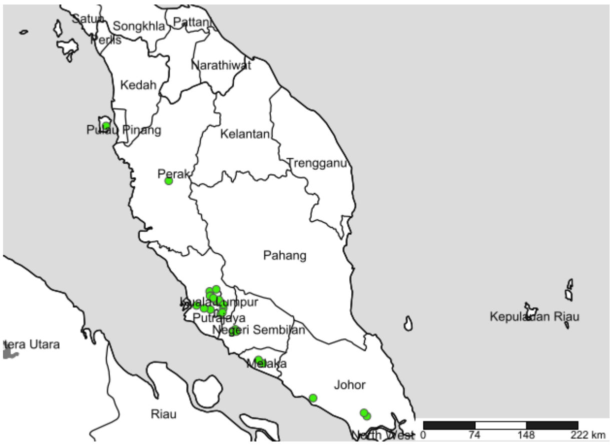


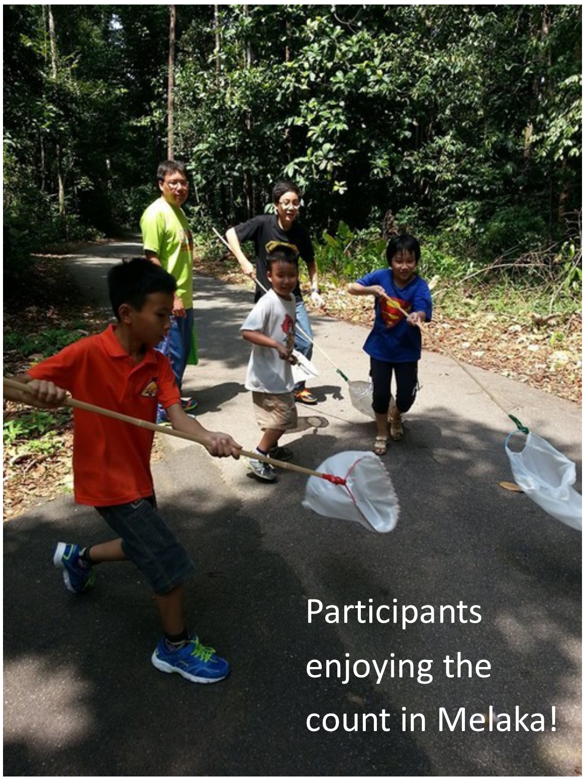


## DID YOU SEE THESE BUTTERFLIES?

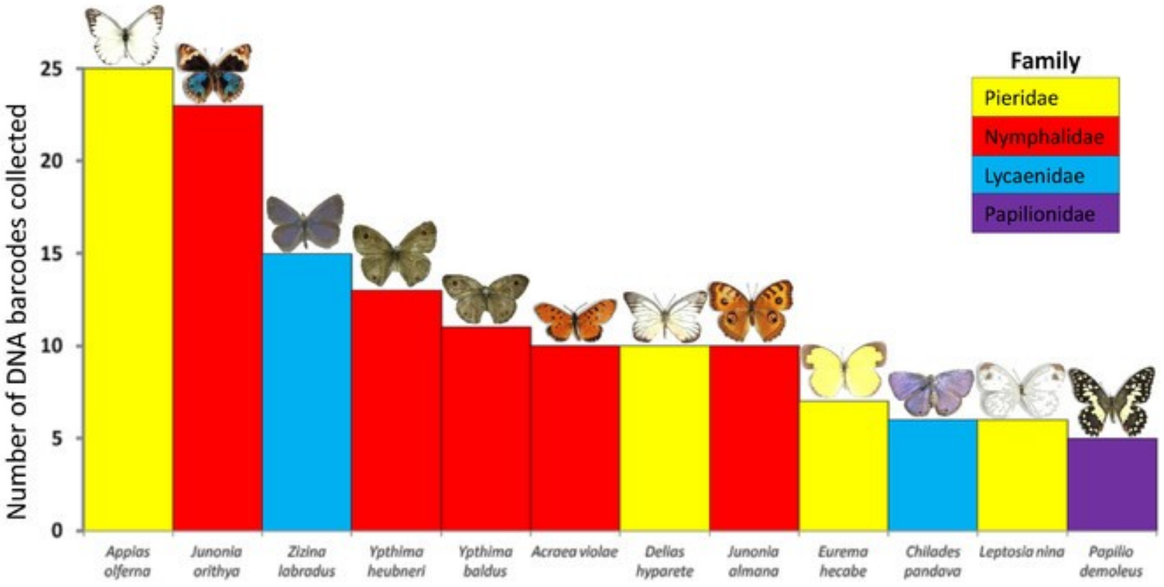


We received 220 butterfly legs and managed to identify 194 of them. This added up to 43 species! The most common species are shown above, but most species were only collected one or two times.

UNIVERSITY OF MALAYA


Volume 3

Issue 4

October 2015


### FIND OUT MORE


Visit us at our website :
**museumzoology.um.edu.my**

Follow us on facebook :
**facebook.com/museumzoologyum**

Contact us by email :
**museumzoology@um.edu.my**


### VISIT US AT UM

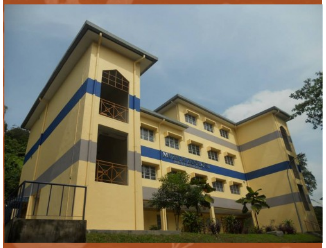


Block F,
Institute of Biological Sciences,
Faculty of Science,
University of Malaya,
50603 Kuala Lumpur


### CONTRIBUTORS


**Peninsular Malaysia Butterfly Count Team**

# DID YOU SEE THESE BUTTERFLIES?

The **most collected butterfly** was

***Appias olferna***

known as the **Striped Albatross**

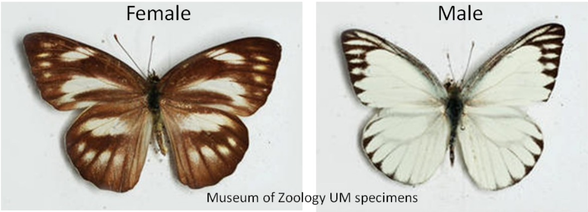

Museum of Zoology UM specimens

The species shows sexual dimorphism which means males and females look different from each other.

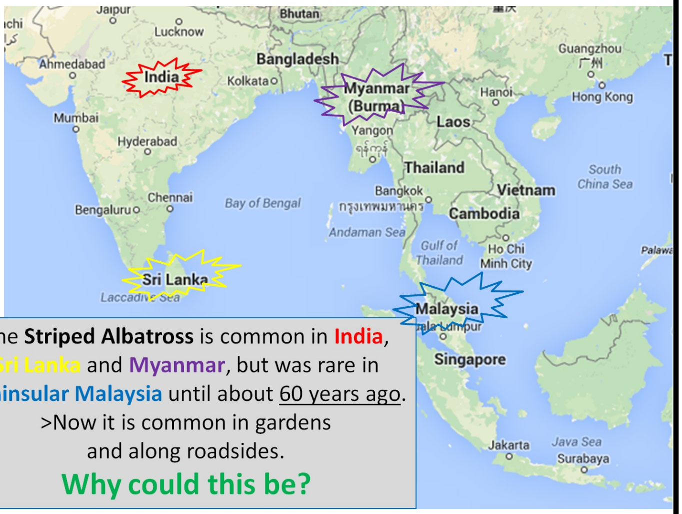


The **Striped Albatross** is common in **India**, **Sri Lanka** and **Myanmar**, but was rare in **Peninsular Malaysia** until about 60 years ago.
>Now it is common in gardens and along roadsides.
**Why could this be?**

Perhaps the **Striped Albatross** has followed its invasive host plant the Fringed Spider Flower into Malaysia?

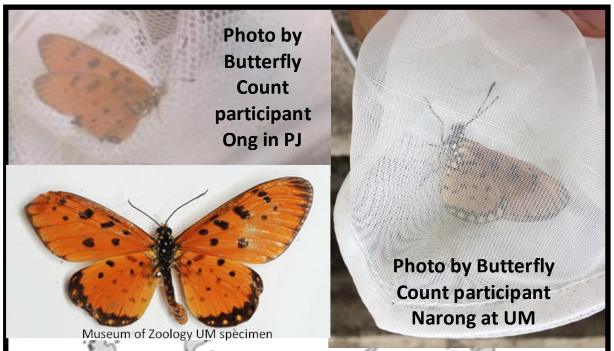


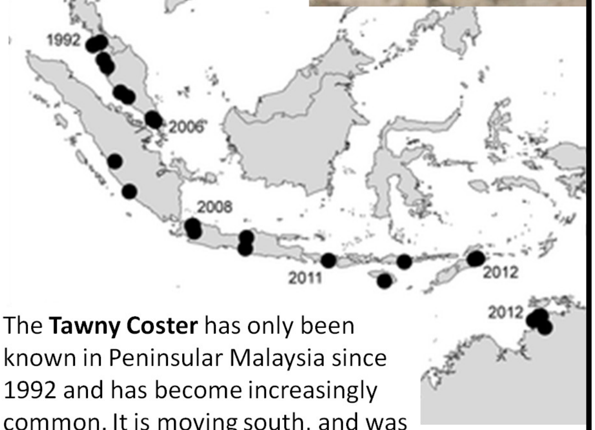


The **Tawny Coster** has only been known in Peninsular Malaysia since 1992 and has become increasingly common. It is moving south, and was reported from Australia in 2012.

Brady et al. 2014

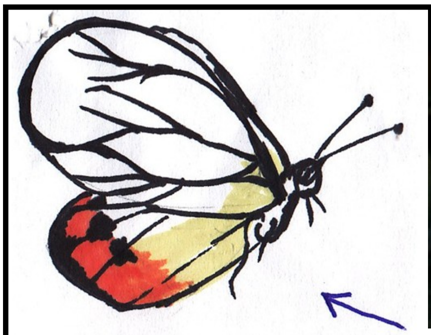
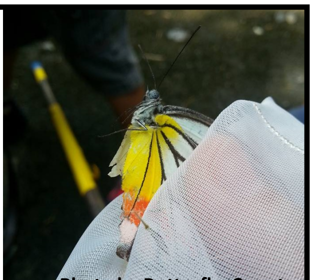

Photo by Butterfly Count participant Yu Cheng Swan

Another common butterfly was *Delias hyparete*, known as the **Painted Jezebel.** We think this is the species seen by Gan Hui Hui in her front yard which she has beautifully illustrated for us.

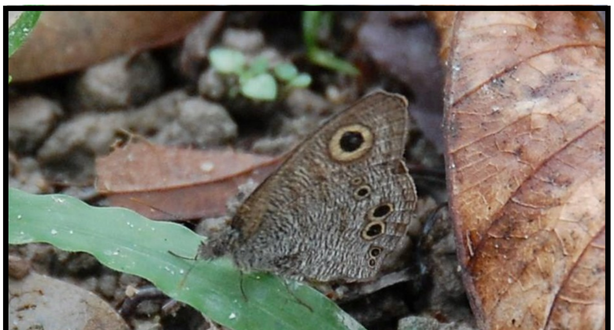

Photo by Butterfly Count participant Dr Nor Azni Abdullah, near Broga, Selangor

The brown **Ring** butterflies include several species in Peninsular Malaysia which all look quite similar. Two **Rings** were among the most collected species. We need to do further research but the DNA barcodes suggest new species for our region may have been collected during the Butterfly Count in Penang!

# MORE INFORMATION?

If you would like to know which species you collected, or more information generally about the result of the Butterfly Count, please send us an email **butterflycount@gmail.com** or give us a call **0379677022 ext. 2115**.
We look forward to hearing from you! Thank you to everyone who took part!
